# Normative FJS-12 scores for the knee in an Asian population: a cross-sectional study

**DOI:** 10.1186/s43019-021-00122-2

**Published:** 2021-10-30

**Authors:** Jia Ying Lee, Wai Weng Yeo, Zi Yang Chia, Paul Chang

**Affiliations:** 1 Department of Orthopaedic Surgery, Singapore General Hospital, 20 College Road, Academia Level 4, Singapore, 169856 Singapore; 2 University of New South Wales, Sydney, Australia

**Keywords:** Normative scores, Patient-reported outcome scores, Forgotten Joint Score, Knee, Sports, Young adults

## Abstract

**Background:**

The Forgotten Joint Score is a patient-reported outcome measure validated in assessing patients post knee arthroplasty, anterior cruciate ligament (ACL) reconstruction surgery and patellar dislocation. A previous study had established the normative scores of a population in the USA but included knees with pathology. The aim of our study is to obtain normative Forgotten Joint Scores in young Asian adults without any pre-existing knee pathologies to increase the interpretability of the Forgotten Joint Score-12 (FJS-12) score.

**Methods:**

We conducted a cross-sectional study across young healthy Asian adults via electronic platforms. Participants who had sought either Western medical consultation, physiotherapy or traditional medical therapies were excluded. Demographic data, occupation, type of sport played, and FJS-12 scores were collected. Scores were stratified into subgroups and analysed.

**Results:**

There were 172 participants who met our inclusion criteria for this study. The average age of participants in our study was 28.1 ± 10.5 years (range 14–70 years), with 83 (47.7%) participants falling into the ages 21–25 years. Average body mass index (BMI) was 21.9 ± 3.3 kg/m^2^ (range 14.7–36.3 kg/m^2^). The average FJS-12 score was 62.8 ± 25.6. The median FJS-12 was 63.5 with a range of 4.2–100. Nine participants (5.2%) scored the maximum score possible, and 56 (32.6%) participants scored below the midpoint score of 50. The percentiles for each subgroup of participants were tabulated and reported. Notably, males aged 46–70 years old scored the highest average FJS-12 score of 73.4 ± 5.5, and females aged 31–45 years old scored the lowest FJS-12 score of 57.1 ± 25.1. Females scored lower than males, although the difference was not statistically significant (*p* = 0.157). There were no significant correlations between BMI, age, or type of sport played with FJS-12; however, interestingly, we observed that women reported similar FJS-12 scores across all age groups, while men reported better scores with increasing age.Interestingly, we observed that women reported similar FJS-12 scores across all age groups, while men reported better scores with increasing age.

**Conclusion:**

Having normative values provides opportunities for benchmarking and comparing individuals against age- and gender-matched peers in the general population. Knowledge of normative values for FJS-12 scores would aid evaluating and tracking progress in patients recovering from injuries or undergoing post-surgery rehabilitation. This would help clinicians  determine if they return to ‘normal’ post intervention.

**Supplementary Information:**

The online version contains supplementary material available at 10.1186/s43019-021-00122-2.

## Background

Patient-reported outcome measures (PROM) are becoming increasingly used tools to determine the effectiveness of surgical intervention in orthopaedic clinical research [1]. While these subjective outcome measures do not fully replace conventional clinical or radiographic parameters, they provide alternative insights into satisfaction, function and quality of life from patients’ perspectives. Recently, goals of health care have shifted towards a more value-oriented approach, and PROMs have been proven to be most suitable for obtaining patient-centric data. The Forgotten Joint Score-12 (FJS-12) is one such PROM that gained popularity after being introduced in 2012 [2]. This score is based on the concept that the primary goal of total knee arthroplasty is for patients to ‘forget’ their artificial joint while performing various everyday tasks. The FJS-12 has been validated in knee arthroplasties, anterior cruciate ligament (ACL) reconstruction surgery and, more recently, in the assessment of patellar dislocation, exhibiting a low ceiling effect and greater sensitivities to marginal differences as compared with other outcome measures [3–5]. PROMs depend on population-specific normative reference ranges to make objective comparisons against. In addition to comparing pre- and post-operative outcomes, these normative ranges also allow clinicians to objectively analyse how close to ‘normal’ patients perceive their joints post-intervention by comparing against a ‘healthy’ control group. A previous study done in the USA has established the normative FJS-12 scores across a general population, but the sampling did not exclude knees with pathology [6]. Therefore, the aim of this study was to obtain FJS-12 scores in young Asian adults without existing knee pathologies to describe the normative percentiles and distribution, to increase the interpretability of FJS-12. We compare our data of normative values with other study populations in the literature.

## Patients and methods

We conducted a cross-sectional study across healthy Asian adults. Participants were invited from an online link via various social media platforms, and the survey was conducted electronically. We calculated a sample size of 177 based on the formula for quantitative variables, using the previously published normative standard deviation for FJS of 34 with a standard normal variate of 1.96, a 5% chance of type 1 error and a precision of 5% [7]. We excluded participants who had sought previous medical consultation, physiotherapy or traditional medicine for prior knee symptoms, regardless of whether a pathology was diagnosed. Demographic data, occupation, type of sport played and FJS-12 scores were collected. The FJS-12 questionnaire was scored using a 5-point Likert scale with raw scores converted into a 0–100-point scale. Higher scores represented better outcomes suggesting the ‘forgotten joint phenomenon’. Normative values for the FJS-12 were presented as mean, standard deviation (SD) and percentiles for the total sample. Age-specific, sex-specific groups and BMI-specific groups based on Asian BMI standards were analyzed. Student’s *t*-test and one-way ANOVA were applied to compare means between groups to test for statistical significance. In addition, we used Pearson's coefficient to investigate  the relationship of FJS-12  with BMI and age.

### Statistical analysis

All statistical analysis was performed with SPSS Statistics for Windows, version 26.0, Armonk, NY: IBM Corp Released 2019. Descriptive and comparative statistical analyses were performed. Variables were compared with either independent *t*-test or one-way ANOVA test depending on the number of groups present. *p* < 0.05 was considered statistically significant, and confidence intervals (CI) were calculated.

## Results

### Demographic data

There were 242 responses, of which 70 responses were excluded for having sought treatment for previous knee symptoms, leaving 172 responses that met our inclusion criteria. The average age of participants in our study was 28.1 ± 10.5 years (range 14–70 years) with 83 (47.7%) participants falling into the age 21–25 years category. Average BMI was 21.9 ± 3.3 kg/m^2^ (range 14.7–36.3 kg/m^2^).

The nature of participants’ occupations fell into three main categories, where students formed the majority (57.4%). Other categories of work were semi-active occupations (sales personnel, events, engineers, healthcare workers) and desk-bound jobs (administrators, bankers). Other than the 14 (8.1%) participants who reported being sedentary, the rest of the participants played a wide variety of sports. The sport being played most often by each participant is represented in Table 1.

**Table 1:** Type of sporting activities and participation. Number of participants shown in brackets, percentages based on a population of 172

Sport participation (172)		*N*	%
Not active (14)		14	8.1
Running (25)		25	14.5
Field sports (33)	Cricket	4	2.3
	Frisbee	1	0.6
	Snowboarding	2	1.2
	Soccer	24	14.0
	Softball	1	0.6
	Touch rugby	1	0.6
Court sports (56)	Badminton	16	9.3
	Basketball	17	9.9
	Floorball	1	0.6
	Netball	11	6.4
	Tennis/squash	7	4.1
	Volleyball	4	2.3
Indoor sports (37)	Bouldering/rock climbing	3	1.7
	Dance	14	8.1
	Gym	8	4.7
	Kickboxing/Muay Thai	8	4.7
	Shooting	1	0.6
	Spin/cycling	1	0.6
	Yoga	2	1.2
Water sports (7)	Swimming	7	4.1

### FJS-12 scores

**Fig. 1:**
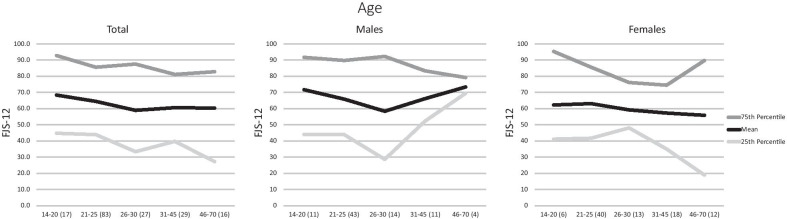
FJS-12 of total population, males and females in different age groups, represented in a line graph. *FJS-12* Forgotten Joint Score-12

**Fig. 2:**
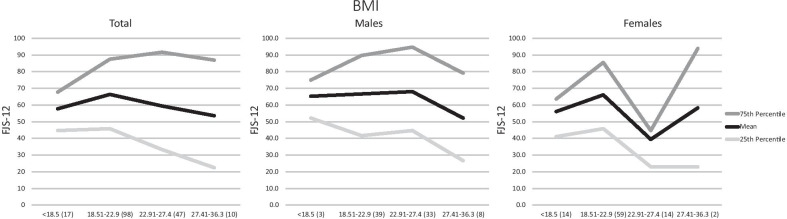
FJS-12 total population, males and females in different BMI ranges, represented in a line graph. *FJS-12* Forgotten Joint Score-12. *BMI* body mass index

The average FJS-12 score was 62.8 ± 25.6 (95% confidence interval for mean 59.0–66.7). The median FJS-12 was 63.5 with a range of 4.2–100. Nine participants (5.2%) scored the maximum score possible, and 56 (32.6%) participants scored below the midpoint score of 50. The percentiles for each subgroup of participants are presented in Table 2. Notably, males aged 46–70 years old scored the highest average FJS-12 score of 73.4 ± 5.5, and females aged 31–45 years old scored the lowest FJS-12 score of 57.1 ± 25.1. Males aged 26–30 years of age showed the largest interquartile range of 63.6. Participants with BMI < 18.5 had the poorest 90th percentile FJS-12 score of 79.6, and females aged 46–70 years old had the poorest 10th percentile FJS-12 score of 10.8 (Figs. 1, 2). The only subgroup to show statistically significant differences were BMI-stratified females, where the best FJS-12 scores were found in females with BMI ranging from 18.51 to 22.9 kg/m^2^. Pearson correlation scores were calculated, and both age and BMI showed a weak negative correlation (−0.107 and −0.085, respectively) with FJS scores.

**Table 2:** Gender, age, occupation and body mass index normative data for FJS-12 knee

		Mean	SD	10th	25th	50th	75th	90th
Total sample (172)		62.8	25.6	27.1	41.7	63.5	85.4	95.8
Gender *p* = 0*.*157	Female (89)	60.1	25.8	25.0	40.6	62.5	84.4	93.7
	Male (83)	65.7	25.2	28.7	43.7	68.7	89.6	95.8
Age (years)*p* = 0*.*706	14–20 (17)	68.2	25.6	32.1	44.8	70.8	92.7	100.0
	21–25 (83)	64.4	24.3	32.1	43.7	64.6	85.4	95.8
	26–30 (27)	58.7	28.7	18.7	33.3	62.5	87.5	95.0
	31–45 (29)	60.5	24.0	27.1	39.6	62.5	81.2	95.8
	46–70 (16)	60.1	30.5	14.2	27.1	69.8	82.8	95.6
Female/age (years) *p* = 0.895	14–20 (6)	62.1	28.7	27.1	41.1	53.1	95.3	100.0
	21–25 (40)	62.9	24.4	31.4	41.7	63.5	85.4	93.5
	26–30 (13)	59.1	24.1	15.0	47.9	62.5	76.0	91.2
	31–45 (18)	57.1	25.1	22.9	34.9	54.2	74.5	98.1
	46–70 (12)	55.8	34.3	10.8	18.7	59.4	89.6	98.1
Male/age (years) *p* = 0.713	14–20 (11)	71.6	24.6	33.7	43.7	83.3	91.7	99.2
	21–25 (43)	65.7	24.4	29.6	43.7	66.7	89.6	95.8
	26–30 (14)	58.3	33.3	15.6	28.6	55.2	92.2	100.0
	31–45 (11)	66.1	22.2	29.6	52.1	62.5	83.3	95.8
	46–70 (4)	73.4	5.5	68.7	69.3	71.9	79.2	81.3
Occupation *p* = 0.293	Students (94)	65.4	24.4	33.3	43.2	67.7	88.0	95.8
	Desk-bound occupation (46)	60.6	25.9	22.2	41.7	60.4	81.8	96.4
	Semi-active occupation (23)	63.8	25.6	24.5	45.8	64.6	85.4	97.5
BMI (kg/m^2^) *p* = 0.204	BMI < 18.5 (17)	57.7	15.8	37.1	44.8	56.2	67.7	79.6
	BMI 18.51–22.9 (98)	66.3	24.3	30.8	45.8	70.8	87.5	95.8
	BMI 22.91–27.4 (47)	59.4	29.4	22.5	33.3	56.2	91.7	95.8
	BMI 27.41–36.3 (10)	53.5	30.0	11.4	22.4	53.1	87.0	93.5
Female/BMI (kg/m^2^) *p* = 0*.*004	< 18.5 (14)	56.1	16.4	36.4	41.1	55.2	63.5	84.4
	18.51–22.9 (59)	66.1	24.3	27.1	45.8	66.7	85.4	93.7
	22.91–27.4 (14)	39.3	27.7	6.2	22.9	33.3	44.8	96.9
	27.41–36.3 (2)	58.3	50.1	22.9	22.9	58.3	93.8	–
Male/BMI (kg/m^2^) *p* = 0.471	< 18.5 (3)	65.3	11.8	52.1	52.1	68.7	75.0	–
	18.51–22.9 (39)	66.5	24.6	31.2	41.7	70.8	89.6	97.9
	22.91–27.4 (33)	68.0	26.0	29.6	44.8	77.1	94.8	95.8
	27.41–36.3 (8)	52.3	28.1	10.4	26.6	53.1	79.1	91.7

*BMI* body mass index. Number of participants shown in brackets for each subgroup. FJS-12: Forgotten Joint Score-12. Variables analysed with either *t*-test or one-way ANOVA, and *p* values represented

A breakdown of scores for individual questions showed that participants noticed their knees least during showering and were most aware of their knees during hiking, climbing stairs and standing for prolonged durations (Table 3). We applied Student’s *t*-test comparing males with females and found no statistically significant difference between scores for individual questions or overall scores. The question with the greatest difference in scores between men and women investigated knee awareness while doing housework or gardening, with females scoring 2.17 as opposed to 2.54 for males (*p* = 0.075, CI −0.786 to 0.038). Cronbach’s coefficient α for all 12 questions was calculated to be 0.94.

**Table 3:** Individual questions in the Forgotten Joint Score-12 and corresponding scores on a scale of 0–4

Question	Score
Are you aware of your knee joint…	
1. … in bed at night	3.12 ± 1.12
2. … when you are sitting on a chair for more than 1 h?	2.70 ± 1.32
3. … when you are walking for more than 15 min?	2.73 ± 1.27
4. … when you are taking a bath/shower?	3.37 ± 0.90
5. … when you are travelling in a car?	2.91 ± 1.19
6. … when you are climbing stairs?	2.09 ± 1.38
7. … when you are walking on uneven ground?	2.47 ± 1.36
8. … when you are standing up from a low-sitting position?	2.12 ± 1.41
9. … when you are standing for long periods of time?	2.10 ± 1.39
10. … when you are doing housework or gardening?	2.35 ± 1.37
11. … when you are taking a walk/hiking?	2.08 ± 1.39
12. … when you are doing your favourite sport?	2.11 ± 1.39

**Fig. 3:**
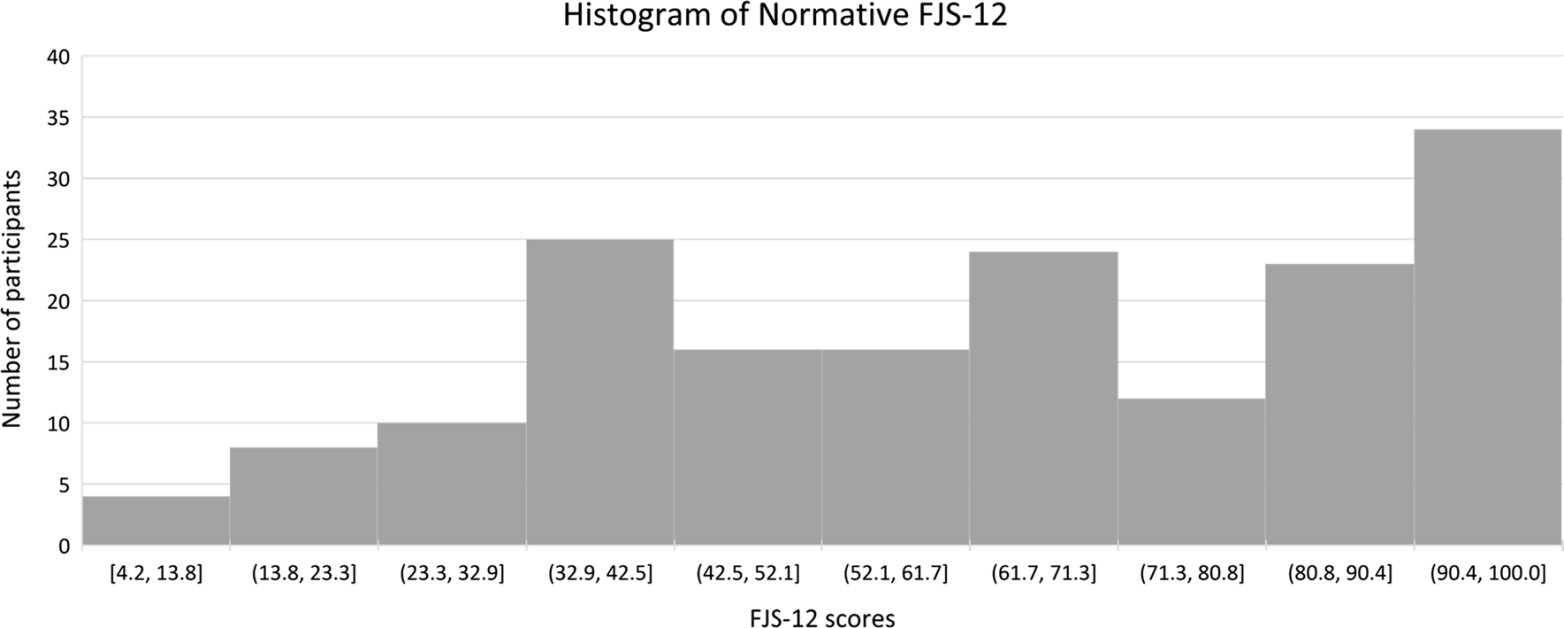
Histogram plot of FJS-12 scores of participants

Kolmogorov–Smirnov test statistic was calculated to be 0.108 (*p* < 0.001), which did not suggest a normal distribution. The distribution curve for FJS-12 scores is shown in Fig. 3.

## Discussion

This was the first study to document normative FJS-12 knee scores of a young Asian population. We presented mean values, age and BMI-stratified values according to gender. The key finding is that, even by surveying participants who had not sought medical treatment for their knees, only a very small number of patients (5.2%) attained the maximum score of 100. A similar study by Giesinger et al. was conducted for a general population in the USA; however, despite their inclusion of knees with pathology, they reported 37.2% of participants attaining maximum scores [6] and an average FJS-12 score of 63.4 ± 35.1(18–39 years old). Behrend et al. analysed FJS-12 scores in patients post-ACL reconstruction against matched healthy control participants [3]. The healthy control group had FJS-12 scores of 88.7 ± 15.0 in a population averaging 31.1 years of age. Another study conducted by Ladurner et al. analysed FJS-12 scores in patients with first-time patellar dislocations against healthy control patients with no previous knee treatment or surgery [4]. His control group had a total FJS-12 score of 87.6 ± 16.6 in a population averaging 29.9 years of age. The lower average FJS-12 scores and fewer participants attaining maximum scores in our study may be attributed to four issues. Firstly, knee osteoarthritis is more prevalent amongst Asians as compared with our Western counterparts as reported by the Beijing osteoarthritis study [8]. Secondly, the Asian population has significantly different cultural habits and functional demands such as sitting cross-legged, squatting and kneeling for chores, hygiene and religious activities, leading to increase loads on the knee [9]. Thirdly, Asians have different thresholds for pain, as reported by Ahn, who noted Asians to have higher risk for clinical pain, heightened sensitivity and depression due to symptomatic knee osteoarthritis [10, 11]. Lastly, cultural differences may play a role in health-seeking behaviours and perception of pain and dysfunction between populations [9].

Interestingly, we observed that women reported similar FJS-12 scores across all age groups, while men reported better scores with increasing age. This is similar to what Giesinger and other previous arthroplasty studies reported, taking into account even those with knee pathology [2, 6]. Bremner-Smith reported other functional knee scores (Oxford Knee Score, Bristol Knee Score, American Knee Society Score) in a normal population, and found a similar negative correlation between age and scores especially when taking into account ‘function’ [12]. We hypothesize that the better scores in older age groups may reflect either an overall decrease in strenuous activities that incite knee symptoms or an improvement in happiness and psychological wellbeing with advancing age [13]. In addition, by excluding participants who had sought medical consultation for knee symptoms, this also causes a selection bias and eliminates those with severe symptoms. In our study, males also reported better scores compared with females for the FJS-12, although not statistically significant. Giesinger agreed that overall, males scored better than females and attributed this to sex selection bias in physical activity patterns [6]. Noyes and Lysholm scores in uninjured athletes have also been reported to be worse in females compared to males [14]. There may be a few explanations for these findings.Firstly, the female gender has been shown to be a risk factor for osteoarthritis of the knee as well as the progression of radiographic changes [15]. Secondly, it has been reported that males typically have higher muscle mass and extensor mechanism strength, which could play a protective role in the biomechanics of the knee [16]. Thirdly, Fillingim demonstrated that females exhibit a greater sensitivity to pain stimuli as compared with males, and since the FJS-12 is based on patient-reported outcomes, this reduces scores further [17, 18]. Additionally, housework is typically performed by women in Asian communities, and this may explain the disparity in scores between genders for that question.

The normative FJS-12 scores did not follow a normal distribution, like other PROMs (Tegner activity scale, Lysholm score, Oxford Knee Score, Knee Society Score, Knee Injury and Osteoarthritis Outcome Score, Western Ontario and McMaster Universities Index) [19, 20]. This finding contrasts with the previously published study of patients post ACL surgery that showed a normal distribution of FJS-12 [21]. We postulate that, if we had included knees with pre-existing pathologies, the scores may have gravitated towards a normal distribution.

### Limitations

Our study has some limitations. Firstly, there may have been participants with knee symptoms but who may not have sought medical attention. This would mean that those who had a high threshold to seek medical attention were included, and this could explain the high number of poor FJS-12 scores we found, especially in the younger age groups. Secondly, the age ranges outside 21–25 years were not as well represented. This may be a reflection of our data collection method. Further studies may be necessary to better define FJS-12 scores across other age groups outside of this range.

## Conclusion

Having normative values provides opportunities for benchmarking and comparing individuals against age- and gender-matched peers in the general population. Knowledge of normative values of FJS-12 scores would aid evaluating and tracking progress in patients recovering from injuries or undergoing post-surgery rehabilitation, to determine if they return to ‘normal’ post intervention.

## Supplementary Information

**Additional file 1.** FJS responses.

## Data Availability

All data generated or analysed during this study are included in this published article and its Additional file 1.

## Abbreviations

PROM: Patient-reported outcome measures
FJS-12: Forgotten Joint Score-12
ACL: Anterior cruciate ligament
BMI: Body mass index
